# Evaluation of Infraorbital Foramen Position Using Cone-Beam Computed Tomography in a Cohort of Central Gujarat, Indian Population

**DOI:** 10.7759/cureus.51722

**Published:** 2024-01-05

**Authors:** Ritvi N Desai, Namish Batra

**Affiliations:** 1 Oral and Maxillofacial Surgery, Manubhai Patel Dental College and Hospital, Vadodara, IND

**Keywords:** premolar, nerve block, incisor, cone-beam computed tomography, alveolar bone

## Abstract

Objective: The location of infraorbital foramen varies between geographic locations. Thus, population-specific information is required to facilitate the prediction of its exact location.

Methods: A cross-sectional study was conducted on 100 cone beam computed tomography (CBCT) sections to evaluate the distance between the infraorbital foramen and incisal edge of the maxillary central incisor, the occlusal plane of the maxillary second premolar, and the alveolar crest over the maxillary second premolar using CBCT in Central Gujarat, Indian population. Descriptive statistical analysis was performed to calculate means and standard deviations for each measured parameter. The statistical significance level was defined at p<0.05.

Results: The distance between the infraorbital foramen and incisal surface of the maxillary central incisor was (mean ± standard deviation) R=49.39 ± 3.12 mm and L=49.49 ± 3.29 mm, the occlusal plane of the maxillary second premolar was R=39.02 ± 2.65 mm and L=39.49 ± 2.53 mm, and the alveolar crest over the maxillary second premolar was R=32.64 ± 2.67 mm and L=31.64 ± 2.33 mm. There was no significant difference in the mean values of all variables between genders and age groups (p>0.05). The distance between the infraorbital foramen and the alveolar crest over the maxillary second premolar was greater on the right side (p<0.05).

Conclusion: The results of this study were analogous to those observed in a cohort of the Turkish population but varied from those observed in a cohort of the Sri Lankan population. Hence, more population-specific studies are required.

## Introduction

The infraorbital foramen (IOF) is located bilaterally within the maxillary bone about 1 cm inferior to the infraorbital margin [1]. The infraorbital nerve (ION), vein, and artery pass through this foramen [2]. The infraorbital nerve is responsible for the sensory innervation to the skin of the malar area between the lower eyelid and the upper lip [1]. The exact anatomic location of the IOF is a crucial landmark in administering an ION block, which aids in managing post-operative pain and the treatment of trigeminal neuralgia [3-5]. An injury to the infraorbital nerve might lead to numbness of the upper lip, lateral wall of the nose, lower lid, and the infraorbital region of the affected side [6]. Furthermore, knowledge of the exact anatomic location of the infraorbital artery (IOA) is critical in various plastic surgery procedures where flaps based on the infraorbital artery are used [7]. The IOA is present in one of the facial danger zones, and injecting dermal fillers in this region may result in arterial occlusion, potentially causing a stroke and blindness [8]. 

The most frequent position of the IOF was found to be in line with the crown of the second premolar [9]. However, several studies in the literature have indicated significant diversity in the shape and placement of the IOF across various populations and ethnic groups, posing potential challenges for numerous surgeons [10]. It is important to highlight that the existence of an accessory infraorbital foramen contributes to the intricacy of this region and necessitates a thorough understanding of the relative position of the IOF [11].

Compared to conventional two-dimensional techniques, cone-beam computed tomography (CBCT) imaging provides reliable data for correct distance measurements. It can obtain detailed information for three-dimensional analysis of the region of interest and provides the main advantage of eliminating the superimposition of neighboring structures [12]. Hardly any information is available on the relative position of IOF in the Indian population. Since population-specific information is important for dental practitioners in the administration of ION block, this study aims to obtain the exact parameters of IOF from the maxillary central incisor and premolars using CBCT to rule out any surgical complications.

## Materials and methods

Study design

This study was carried out after obtaining clearance from the institutional ethical committee of Manubhai Patel Dental College (approval number: MPDC_264/OS-01-23) between March 2023 and August 2023. A descriptive cross-sectional analysis was conducted on a sample of 100 CBCT sections belonging to healthy Indian patients (Central Gujarat population). CBCT images of patients above 18 years of age, demonstrating a clear image of the maxillary central incisors and second premolars, devoid of gross malocclusions, and periodontal pathologies, were included in the analysis. In addition, images belonging to patients having craniofacial anomalies, such as cleft lip and palate or bone pathologies, maxillofacial trauma, grossly carious teeth, missing teeth, root stumps, and impacted teeth, were excluded.

CBCT image analysis

**Figure 1:**
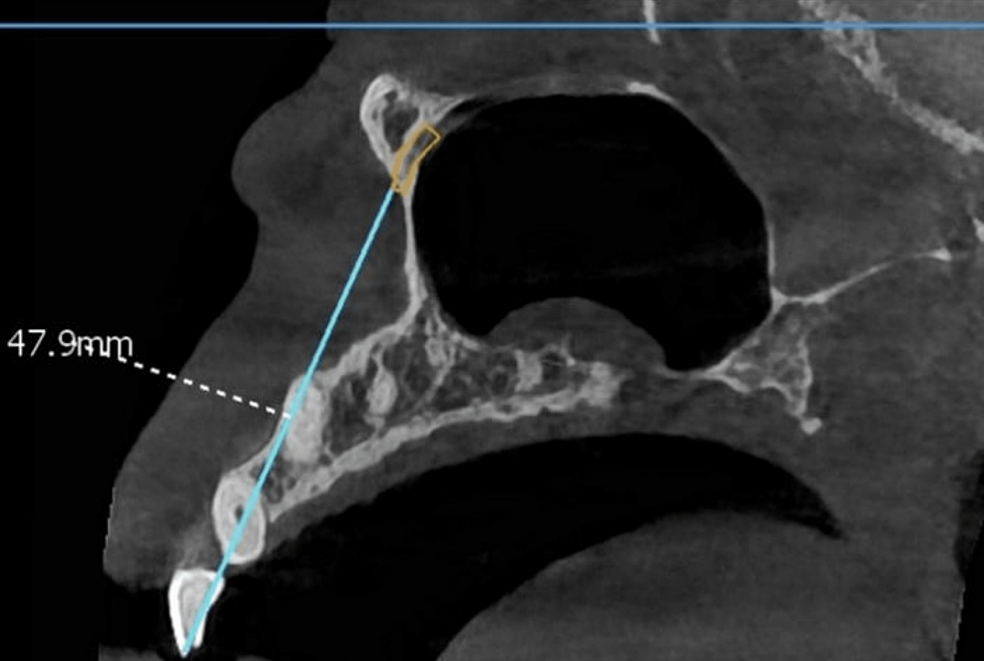
Distance between the infraorbital foramen and the incisal edge of the maxillary central incisor (point A) was measured in sagittal cross-section.

Hundred CBCT sections were acquired from Smart Scan Centers (Vadodara, Central Gujarat) using the VATECH Smart Plus imaging system (Vatech Co., Hwaseong, Korea) with the following exposure parameters: 99 kVp, 12 mA, 16.4 s exposure time, and a field-of-view (FOV) of 10 x 8 cm. The CBCT sections were analyzed as de-identified digital imaging and communications of medicine (DICOM) using EzScan Software (Vatech Co., Hwaseong, Korea). Two calibrated observers interpreted the CBCT scans, and the mean of their observations was derived. The evaluation of the distance between the IOF and the incisal edge of the maxillary central incisor (point A) was done in the sagittal cross-section (Figure 1).

**Figure 2:**
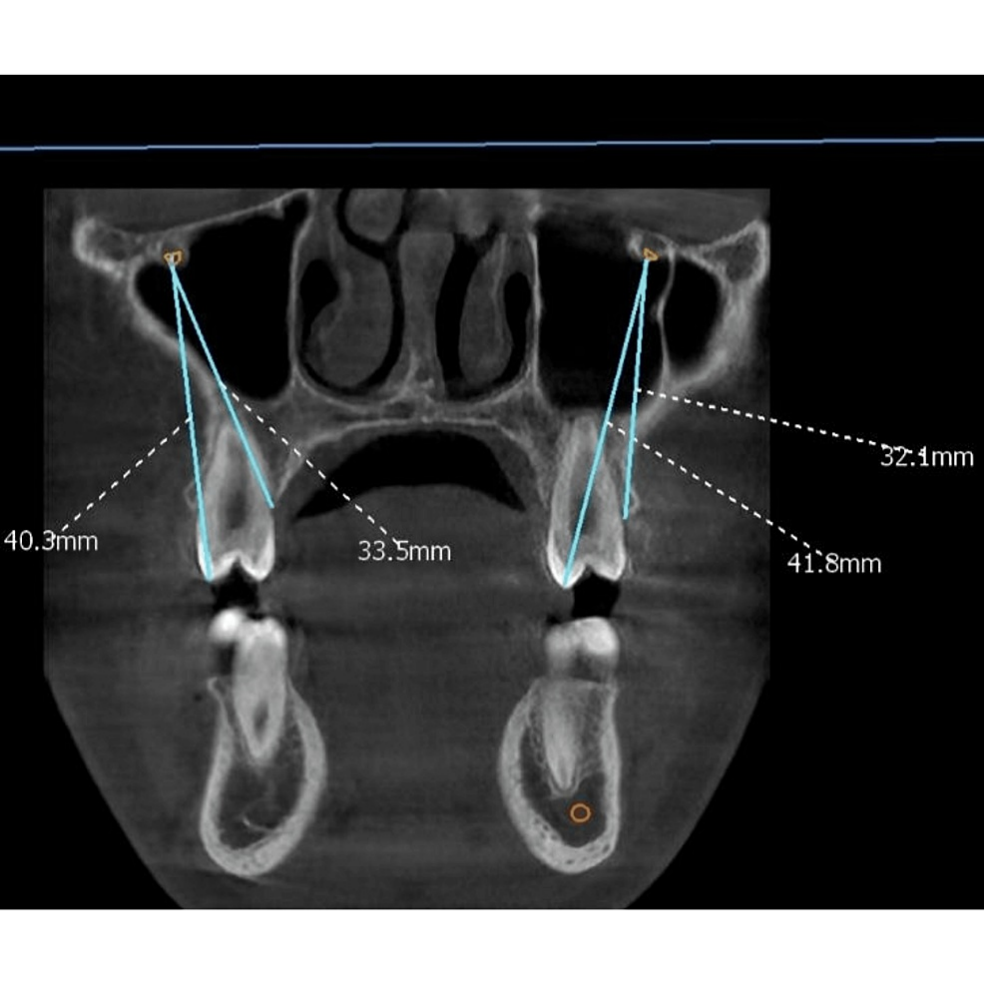
The distance between the infraorbital foramen and the occlusal plane of the maxillary second premolar (point B) and the mesial alveolar crest over the maxillary second premolar (point C) was measured in the coronal cross-section.

The distance between the IOF and the occlusal plane of the maxillary second premolar (point B) and the mesial alveolar crest over the maxillary second premolar (point C) was evaluated in the coronal cross-section (Figure 2).

Statistical analysis

All statistical analyses were performed using STATA/IC-13 (StataCorp LP, College Station, Texas, USA). The Kolmogorov-Smirnov test and box plots were used to assess whether the quantitative variables followed a normal distribution, and further analysis of these variables was done using parametric tests. An independent t-test was used to compare the mean values of all variables between genders. A paired t-test was performed for the comparison of numeric variables between the right and left sides. A one-way analysis of variance (ANOVA) was used to compare the mean values of all variables between age groups. Parametric data were expressed as the mean and standard deviation. The statistical significance level was defined at p<0.05.

## Results

This study examined the CBCT images of 100 patients (26% males and 74% females), whose ages varied between 18 and 72 years old (mean ± standard deviation, 41.06 ± 14.81 years), meeting the inclusion criteria. 

The following distances were measured from the IOF to the anatomic landmarks: (a) The mean distance from the IOF to the incisal edge of the maxillary central incisor (point A) was 49.39 ± 3.12 mm and 49.49 ± 3.29 mm on the right and left sides, respectively. (b) The mean distance from the IOF to the occlusal plane of the maxillary second premolar (point B) was 39.02 ± 2.65 mm and 39.49 ± 2.53 mm on the right and left sides, respectively. (c) The mean distance from the IOF to the mesial alveolar crest over the maxillary second premolar (point C) was 32.64 ± 2.67 mm and 31.64 ± 2.33 mm on the right and left sides, respectively (Table 1).

**Table 1:** Mean values of the distances from the infraorbital foramen to the anatomic landmarks. The data has been represented as mean ± SD, and the statistical significance level is defined at p<0.05.

Anatomic landmark	Mean	SD	p-value
Right second premolar	39.092	2.6524	0.114
Right alveolar crest	32.644	2.6711	0.200
Right central incisor	49.392	3.1293	0.200
Left second premolar	39.638	2.5305	0.200
Left alveolar crest	31.664	2.3357	0.200
Left central incisor	49.498	3.2950	0.079

When these distances were compared for genders using an independent t-test, no statistical difference was identified (p>0.05) (Table 2). This indicates that the mean distances from the IOF to the anatomic landmarks were similar between males and females.

**Table 2:** Comparison of mean between genders. The data has been represented as mean ± SD, and the statistical significance level is defined at p<0.05.

	Gender	Mean	SD	p-value
Right second premolar	Male	39.992	2.20	0.157
	Female	38.776	2.74	
Right alveolar crest	Male	33.308	2.19	0.302
	Female	32.411	2.80	
Right central incisor	Male	50.423	2.91	0.170
	Female	49.030	3.16	
Left second premolar	Male	40.038	1.94	0.513
	Female	39.497	2.71	
Left alveolar crest	Male	31.969	2.11	0.589
	Female	31.557	2.42	
Left central incisor	Male	50.715	3.86	0.123
	Female	49.070	3.01	

As shown in Table 3, one-way analysis of variance (ANOVA) was used to compare the mean distances from the IOF to the anatomic landmarks between age groups. No statistical difference was found in the mean distance from the IOF to the anatomic landmarks (p>0.05), which suggests that the mean distances from the IOF to the anatomic landmarks are similar between different age groups.

**Table 3:** Comparison of mean between age groups. The statistical significance level is defined at p<0.05.

Variables	Source of variation	Sum of squares	df	Mean square	F-value	p-value
Right second premolar	Between groups	48.540	5	9.708	1.442	0.228
	Within groups	296.196	44	6.732		
	Total	344.737	49			
Right alveolar crest	Between groups	35.287	5	7.057	0.988	0.436
	Within groups	314.316	44	7.144		
	Total	349.603	49			
Right central incisor	Between groups	30.907	5	6.181	0.606	0.696
	Within groups	448.930	44	10.203		
	Total	479.837	49			
Left second premolar	Between groups	24.775	5	4.955	0.754	0.587
	Within groups	289.003	44	6.568		
	Total	313.778	49			
Left alveolar crest	Between groups	43.875	5	8.775	1.728	0.148
	Within groups	223.440	44	5.078		
	Total	267.315	49			
Left central incisor	Between groups	47.225	5	9.445	0.857	0.517
	Within groups	484.764	44	11.017		
	Total	531.990	49			

When these distances were compared between the right and left sides using a paired t-test, the distance between the IOF and point C was found to be greater on the right side compared to the left side (p<0.05), and the distance from the IOF to points A and B was almost equal on both sides (p>0.05) (Table 4).

**Table 4:** Comparison of the mean between the right and left sides. The data has been represented as mean ± SD, and the statistical significance level is defined at p<0.05.

Variable	Side	Mean	SD	p-value
Second premolar	Right	39.092	2.65	0.131
	Left	39.638	2.53	
Alveolar crest	Right	32.644	2.67	0.006
	Left	31.664	2.33	
Central incisor	Right	49.392	3.12	0.725
	Left	49.498	3.29	

## Discussion

The infra-orbital nerve block is achieved by depositing the local anesthetic agent at the exit of the infra-orbital nerve from the infra-orbital foramen, either by the ‘central incisor approach’ or the ‘bicuspid approach’ [13]. To the best of our knowledge, no other study has evaluated the distance between the IOF and the incisal edge of the maxillary central incisor. In this study, the mean distance between IOF and point A was R=50.42 ± 2.91 mm and L=50.71 ± 3.86 mm in males and R=49.03 ± 3.16 mm and L=49.07 ± 3.01 mm in females. This knowledge of the exact location of the IOF from the incisal edge of the maxillary central incisor is beneficial during the administration of the ION block through the central incisor approach.

Also, the mean distance between the IOF and point B in this study was R=39.92 ± 2.20 mm and L=40.03 ± 1.94 mm in males and R=38.76 ± 2.70 mm and L=39.49 ± 2.71 mm in females. This is analogous to a study conducted by Bahşi et al. [14] in a cohort of a Turkish population of 75 female and 75 male subjects aged 18-65 years, where the distance between the IOF and the occlusal plane of the second premolar tooth was R=39.33 ± 2.99 mm and L=39.94 ± 3.06 mm and R=38.04 ± 1.75 mm and L=37.94 ± 1.96 mm in females, and no statistical difference was found between genders. However, in a study conducted by Raschke et al. [15], where spiral computed tomography (CT) scans were reviewed, the average distance between the IOF and the cusp tip of the second premolar was 41.81 ± 1.07 mm and 37.33 ± 1.58 mm in males and females, respectively (Table 5). This suggests that the distance from the IOF to anatomic landmarks may vary when different imaging modalities are used. This population-specific information of the exact location of the IOF from the occlusal plane of the maxillary second premolar is beneficial during the administration of the ION block through the bicuspid approach.

**Table 5:** Comparison of the distance from the infraorbital foramen to the occlusal plane of the maxillary second premolar with other studies. The data has been represented as mean ± SD.

	Male		Female
Our study	R	R=39.92 ± 2.20 mm	38.76 ± 2.70 mm
	L	40.03 ± 1.94 mm	39.49 ± 2.71 mm
Bahşi et al. [14]	R	39.33 ± 2.99 mm	38.04 ± 1.75 mm
	L	39.94 ± 3.06 mm	37.94 ± 1.96 mm
Raschke et al. [15]	41.81 ± 1.07 mm		37.23 ± 1.58 mm

Analyzing dry skulls in an Indian population, Aggarwal et al. [5] reported the mean distance from IOF to point C as 28.41 ± 2.82 mm. The distance from IOF to point C in our study was R=33.30 ± 2.19 mm and L=31.96 ± 1.94 mm in males and R=32.41 ± 2.71 mm and L=31.55 ± 2.71 mm in females, which is greater than the distance evaluated in the above-mentioned study (Table 6). This can be due to the variability of the methods used in each study. In a study conducted by Thilakumara et al. [6] in a cohort of the Sri Lankan population, the mean distance from the IOF to the crest of the alveolar bone to the mid-point of the IOF was 29.59 ± 3.59 and 29.65 ± 3.28 on the right and left sides, respectively, which is less than the distance ascertained in this study. This suggests significant diversity in the location of IOF among distinct populations.

**Table 6:** Comparison of the distance from the infraorbital foramen to the alveolar crest over the maxillary second premolar with other studies. The data has been represented as mean ± SD.

	Male		Female
Our study	R	33.30 ± 2.19 mm	32.41 ± 2.71 mm
	L	31.96 ± 1.94 mm	31.55 ± 2.71 mm
Thilakumara et al. [6]	R	29.60 ± 3.66 mm	29.58 ± 2.94 mm
	L	29.85 ± 3.48 mm	29.46 ± 2.48 mm

Very few other studies have attempted to evaluate the morphometric characteristics of the IOF using CBCT [6,14,16,17]. In a study conducted by Sokhn et al. on the Lebanese population, the distance from the IOF to the infraorbital margin was 7.98 ± 1.41 mm, the mean distance between the IOF and the lateral nasal wall was 10.61 ± 2.39 mm, and the mean distance between the IOF and the middle of the face was 24.71 ± 2.09 mm. A statistical difference was identified with respect to gender. Also, 54.8% of the IOF were circular, and the mean diameter of the foramina was 3.71 ± 0.63 mm, which did not show any statistically significant difference in all age groups [16]. Ali et al. [9], Ilayperuma et al. [18], and Zdilla et al. [19] reported the presence of IOF above the vertical axis of the second premolar. However, in a study conducted in a cohort of Cameroonian adults, the IOF was located at the line passing below the maxillary first molar [17]. Two separate infraorbital canals with ION that opened to the anterior surface of the maxilla were identified by Leo et al. [20] in a cadaver.

No other studies, to the best of our knowledge, have been conducted within Central Gujarat, Indian population. The IOF shows a lot of variability between different populations. These differences in the morphometric characteristics of the IOF may be due to racial differences and the variability of the methods used in each study. However, it is crucial to acknowledge limitations like a relatively small sample size and age-related variations that may impact the generalizability and interpretation of our results. The future scope of this study is to mitigate these limitations by carrying out further population- and age-specific studies with a larger sample size and evaluating the morphometric characteristics of accessory foramen. 

The knowledge of the population-specific information location of the IOF has various clinical implications, such as reducing the rate of failure of the ION block, in surgical planning for Orthognathic Surgery, in Open Reduction and Internal Fixation (ORIF) of LeFort [21] and zygomaticomaxillary complex fractures, and forensic sciences.

## Conclusions

In conclusion, we suggest that the parameters found in the present study may aid in the prediction of the location of IOF in the Indian population, as no other studies have evaluated the position of IOF using CBCT in this population. The results of this study were analogous to those observed in a cohort of the Turkish population but varied from a cohort of the Sri Lankan population. Hence, we suggest that further population- and age-specific studies should be conducted in the future.

Informed parameters regarding the knowledge and location of the infraorbital foramen can significantly enhance surgical treatment planning, in turn decreasing the risk of ION damage during various maxillofacial and esthetic surgeries and holding promise for an improved success rate of ION block.
